# The current status and influencing factors of medication literacy among patients with cardiovascular diseases: a systematic review and meta-analysis

**DOI:** 10.3389/fphar.2026.1849302

**Published:** 2026-06-03

**Authors:** Ting Xu, Qian Yao, Yu Ni, Xiuchuan Li

**Affiliations:** 1 College of Nursing, Chengdu University of Traditional Chinese Medicine, Chengdu, China; 2 Department of Emergency, The Third People’s Hospital of Chengdu, Chengdu, China; 3 School of Nursing, Chengdu Medical College, Chengdu, China; 4 Department of Cardiology, General Hospital of the Western Theater of the Chinese People’s Liberation Army, Chengdu, China

**Keywords:** cardiovascular diseases, medication literacy, health ecological model, influencing factors, meta-analysis

## Abstract

**Background:**

As an important domain of health literacy, medication literacy plays a critical role in ensuring the safe and effective use of medicines, especially among individuals with Cardiovascular Diseases (CVDs). Nevertheless, evidence regarding the overall level of medication literacy and the factors influencing it in this population remains limited. This study aimed to systematically evaluate medication literacy levels among patients with CVDs and to identify associated factors based on the Health Ecological Model (HEM).

**Methods:**

This systematic review and meta-analysis was conducted in accordance with the Preferred Reporting Items for Systematic Reviews and Meta-Analyses (PRISMA) statement. We systematically searched PubMed, Web of Science, the Cochrane Library, Embase, China National Knowledge Infrastructure (CNKI), Wanfang Data, and the Vertically Integrated Projects database (VIP) for studies published in English or Chinese from database inception to 30 December 2025. Two reviewers independently performed study selection, quality assessment, data extraction, and analysis. Mean medication literacy scores and corresponding effect sizes were pooled using meta-analysis in Stata 18.0. Random-effects models were applied for all analyses due to substantial heterogeneity across studies. Heterogeneity was assessed using the I^2^ test. Sensitivity analyses and publication bias were also assessed.

**Results:**

A total of 34 studies involving 9,599 patients were included. Of these, 30 studies reported medication literacy levels. Given the substantial heterogeneity observed across all instrument subgroups, random-effects models were applied for pooling. The pooled mean scores were as follows: the Medication Literacy Questionnaire (MLQ): 4.56 [95% CI (4.36, 4.77), I^2^ = 95.7%]; the Medication Literacy Scale in Spanish and English (MedLitRxSE): 7.49 [95% CI (6.46, 8.52), I^2^ = 97.7%]; and the Chinese Medication Literacy Scale for Hypertensive Patients (C-MLSHP): 24.09 [95% CI (23.72, 24.45), I^2^ = 64.2%]. Twenty-four studies reported factors associated with medication literacy. Significant factors included age, department of hospitalization, comorbid hypertension, and number of discharge medications (personal traits); self-efficacy (psychological and behavioral characteristics); social support (social networking); income level and educational attainment (work and living conditions); and receipt of medication education (policy and environmental factors).

**Conclusion:**

Medication literacy among patients with CVDs remains suboptimal and is influenced by multidimensional factors. Individualized patient education, standardized counseling protocols, community-based services, and healthcare provider training are urgently needed. Multilevel interventions grounded in the HEM hold promise for improving medication use and informing policy development. Future large-scale, longitudinal studies employing standardized assessment tools are needed to elucidate causal relationships, underlying mechanisms, and sources of heterogeneity.

**Systematic Review Registration:**

The protocol for this systematic review has been registered in PROSPERO (CRD420251208289, available at: https://www.crd.york.ac.uk/PROSPERO/view/CRD420251208289).

## 1 Introduction

Cardiovascular diseases (CVDs) encompass a broad range of conditions affecting the heart and blood vessels, including coronary heart disease, cerebrovascular disease, peripheral arterial disease, rheumatic heart disease, congenital heart disease, deep vein thrombosis, and pulmonary embolism (Oliveira et al., 2024). CVDs continue to be the primary contributor to global mortality. According to the World Health Organization, approximately 19.8 million deaths were attributed to CVDs in 2022, accounting for 32% of all global deaths, and this number is projected to rise to 23.6 million by 2030 (World Health Organization, 2025). Pharmacotherapy remains central to CVD management. Antihypertensive agents, antiplatelet drugs, statins, and beta-blockers are well established in reducing morbidity and mortality (McEvoy et al., 2024). Despite these advances, the effectiveness of these therapies is closely tied to patients’ ability to understand and adhere to prescribed regimens. In clinical practice, inappropriate medication use remains common and contributes to poor health outcomes and increased healthcare costs (Rasmussen et al., 2007).

Medication literacy is generally regarded as a specific extension of health literacy that applies to medication-related contexts (Santana et al., 2021). It describes how individuals access, interpret, and act upon information related to their medications in order to make appropriate decisions (Pouliot et al., 2018). Patients with CVDs often require long-term and complex pharmacotherapy, and inadequate medication literacy can result in misinterpretation, dosing errors, and a greater likelihood of adverse events (Custodis et al., 2016). Existing evidence indicates that low medication literacy is associated with poor medication adherence, higher rates of hospital readmission, and a greater occurrence of adverse drug events (Kolandaivelu et al., 2014).

Medication literacy has received growing attention; yet, the overall level of medication literacy among patients with CVDs worldwide remains unclear. In addition, variations in sample sizes, measurement instruments, study populations, and geographic regions contribute to substantial discrepancies in medication literacy levels across studies. Current evidence lacks a coherent synthesis of evidence regarding the factors influencing medication literacy. This heterogeneity in the existing evidence hinders the development of targeted medication management interventions. To better capture the factors influencing medication literacy among patients with CVDs, the application of theoretical frameworks becomes particularly useful. The Health Ecological Model (HEM) is one of the most widely used frameworks for explaining health-related behaviors, including patients’ medication management behaviors (Diab et al., 2018; Sun et al., 2020). According to the HEM, health behaviors are shaped by multilevel factors arising from interactions across five levels: personal traits, psychological and behavioral characteristics, social networking, work and living conditions, and policy and environmental factors (Institute of Medicine US Committee on Educating Public Health Professionals for the 21st Century et al., 2003). Although previous studies have examined medication literacy in specific cardiovascular populations, a comprehensive quantitative synthesis remains lacking. In particular, no prior meta-analysis has systematically integrated both medication literacy levels and their influencing factors within a unified theoretical framework. Therefore, this study not only quantifies medication literacy across different measurement tools, but also applies the HEM to organize and analyze multilevel determinants, thereby providing a more structured and theory-driven understanding of this issue.

## 2 Materials and methods

This systematic review and meta-analysis were conducted and reported in accordance with the Preferred Reporting Items for Systematic Reviews and Meta-Analyses (PRISMA) guidelines. As all data were obtained from publicly available databases, no additional ethical approval or patient consent was required. The protocol was prospectively registered in the PROSPERO database prior to study initiation (registration No.: CRD420251208289).

### 2.1 Literature retrieval strategy

We systematically searched PubMed, Web of Science, the Cochrane Library, Embase, China National Knowledge Infrastructure (CNKI), Wanfang Data, and the Vertically Integrated Projects (VIP) database for cross-sectional or cohort studies investigating medication literacy and its associated factors among patients with CVDs. We also screened the reference lists of relevant studies and searched the grey literature. The search period spanned from database inception to 30 December 2025. The search was conducted by combining subject words and free words and adjusting them according to the characteristics of each database. The search terms included terms related to “cardiovascular disease,” “cerebrovascular disease,” “coronary heart disease,” “stroke,” “heart failure,” and “hypertension,” as well as “medication literacy,” “medication use literacy,” and “pharmaceutical literacy.” Detailed search strategies for each database are provided in the Supplementary Material.

### 2.2 Inclusion and exclusion criteria

Inclusion criteria: ① Population: patients diagnosed with CVDs; ② Research theme: medication literacy levels and/or associated factors; ③ Study design: cohort, case‐control, or cross-sectional studies; ④ Outcome indicators: medication literacy scores; strength of association between each risk factor and medication literacy (SMD and correlation coefficient r, with 95% CI); ⑤ For duplicate publications, the study with higher methodological quality was included if the core authors, institutions, and reported results were consistent.

Exclusion criteria: ① Studies not published in English or Chinese; ② Studies with unavailable full texts or incomplete data; ③ Studies assessed as low quality; ④ Reviews, conference abstracts, patents, and other non‐original studies.

### 2.3 Literature screening and data extraction

All retrieved records were imported into EndNote 21 software, and duplicates were removed. Two reviewers independently screened the studies. First, titles and abstracts were screened to exclude irrelevant studies; subsequently, the full texts of potentially eligible studies were assessed according to the predefined inclusion and exclusion criteria. Disagreements between the two reviewers were resolved through discussion or consultation with a third reviewer. Data were extracted from the included studies, including first author, publication year, study design, sample size, medication literacy scores, measurement tools, and main associated factors.

### 2.4 Quality assessment of literature

Two systematically trained reviewers independently assessed the methodological quality of the included cross-sectional studies using the evaluation criteria recommended by the Agency for Healthcare Research and Quality (AHRQ) (Zeng et al., 2012). The total possible score was 11 points, with scores ranging from 0 to three indicating low quality, four to seven indicating moderate quality, and 8 to 11 indicating high quality.

### 2.5 Statistical analysis

Statistical analyses were performed using Stata version 18.0. Raw mean scores and corresponding standard deviations (SDs) or standard errors (SEs) were used as the primary effect measures. To avoid methodological concerns associated with cross-instrument pooling, all meta-analytic pooling of raw mean scores was performed strictly within instrument-specific subgroups, and no raw mean scores were combined across instruments with different scoring ranges or conceptual frameworks; the results are presented as weighted mean values with 95% confidence intervals (CIs). Prior to pooling correlation coefficients for medication literacy, studies reporting correlation coefficients (r) were transformed using Fisher’s Z transformation, and corresponding SEs were calculated. The pooled Fisher’s Z values were then obtained and subsequently converted back to pooled correlation coefficients (r). Heterogeneity was assessed using the chi-square test and the I^2^ statistic. A fixed-effects model was used when P ≥ 0.10 and I^2^ ≤ 50%; otherwise, a random-effects model was applied, and potential sources of heterogeneity were further explored. Sensitivity analyses were conducted to evaluate the robustness of the results. Publication bias was assessed using Egger’s test, and a P-value < 0.05 was considered statistically significant.

## 3 Results

### 3.1 Results of literature searching

**FIGURE 1:**
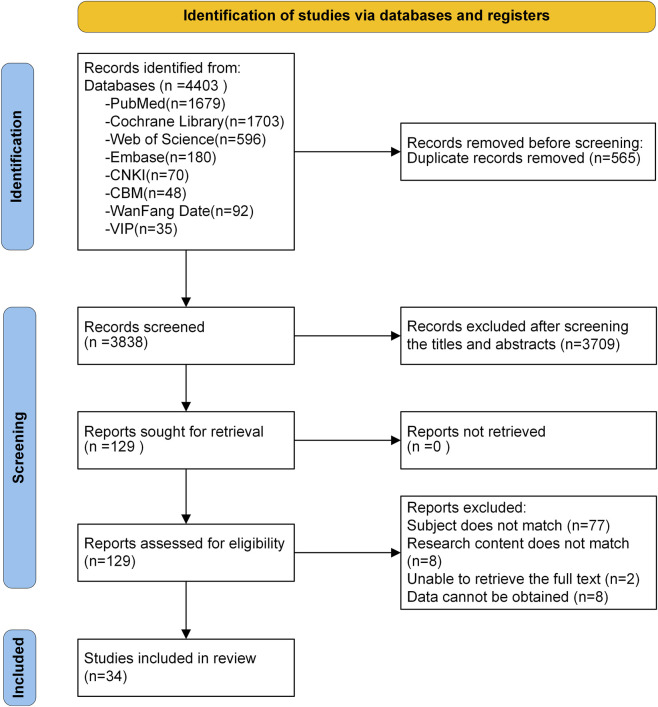
PRISMA flow diagram of the study selection process.

A total of 4,403 records were identified through database searching. After removing duplicates, 3,838 records remained. Following title and abstract screening, 3,709 records were excluded. The full texts of 129 articles were assessed for eligibility, of which 34 studies were ultimately included in the final analysis, involving a total of 9,599 participants. The literature screening process is shown in Figure 1.

### 3.2 Basic characteristics and quality evaluation of the included studies

All 34 included studies were cross-sectional in design, published between 2016 and 2026, with a total sample size of 9,599 participants. The studies were conducted in China and Indonesia. The methodological quality of the included studies was assessed, with scores ranging from 5 to 9; twelve studies were rated as high quality, and twenty-two as moderate quality. Detailed characteristics of the included studies and quality assessment results are presented in Table 1.

**TABLE 1:** Basic characteristics and quality assessment of the included literature.

First author	Year	Diseases type	Country	Study design	Measure tool	Sample size	Average mean score ( $\bar{x}$ ±s)	Influencing factor	Quality score (AHRQ)
Yingli xu	2021	Acute myocardial infarction	China	Cross-sectional	A	83	3.54 ± 0.87	1 3 4	6
Fan nie	2024	Atrial fibrillation	China	Cross-sectional	A	154	4.18 ± 2.00	2 3 5	6
Yueping wang	2021	Coronary heart disease	China	Cross-sectional	A	150	4.73 ± 1.37	6 7 8	6
Jieli ye	2024	Coronary heart disease	China	Cross-sectional	A	100	−	6 7 8	5
Ao jiao	2023	Coronary heart disease	China	Cross-sectional	A	107	4.66 ± 1.01	1 3 9 10 11 12	7
Jialing Li	2023	Cardiovascular disease	China	Cross-sectional	F	486	111.00 ± 12.36	1 4 12 13	8
Chuantao xie	2024	Chronic heart failure	China	Cross-sectional	B	412	8.38 ± 4.60	−	7
Feng zheng	2017	Acute coronary syndrome	China	Cross-sectional	A	253	4.75 ± 1.42	1 3	7
Nana du	2023	Stroke	China	Cross-sectional	A	287	4.58 ± 0.32	−	7
Junkun ban	2020	Discharge after PCI	China	Cross-sectional	A	114	3.94 ± 1.66	1 3 4 14	6
Rui zhang	2024	Stroke	China	Cross-sectional	A	316	5.03 ± 1.60	−	7
Zhenfeng chen	2024	Stroke	China	Cross-sectional	A	212	5.52 ± 1.63	3 5 15	5
Mingjun zhang	2025	Coronary heart disease	China	Cross-sectional	B	195	6.19 ± 3.53	−	6
Ying yao	2025	Stroke	China	Cross-sectional	E	285	48.26 ± 12.51	3 4 10 16 17	7
Zishan huang	2025	Atrial fibrillation	China	Cross-sectional	A	166	4.46 ± 1.09	3 4 6 8 9 11 12 18	8
Yan han	2023	Stroke	China	Cross-sectional	B	400	8.78 ± 3.51	1 3 6 19 20	5
Kunxiu zhang	2021	Hypertension	China	Cross-sectional	A	338	3.00 (3.00, 4.00)	−	7
Linlin hou	2024	Stroke	China	Cross-sectional	A	278	4.79 ± 1.75	4 6 8 21	8
Lixiang zhang	2019	Atrial fibrillation	China	Cross-sectional	A	106	4.67 ± 1.03	3 9 11 12 22	8
Lili hao	2018	Cardiovascular disease	China	Cross-sectional	B	200	7.60 ± 2.98	1 3 23	5
Ning qin	2024	Hypertension	China	Cross-sectional	D	378	28.44 ± 8.78	−	8
Shuangjiao shi	2019	Hypertension	China	Cross-sectional	C	420	24.03 ± 5.13	−	8
Li qiao	2021	Coronary heart disease	China	Cross-sectional	B	416	4.96 ± 4.68	2 3 4 17 24	6
Zhiying shen	2022	Hypertension	China	Cross-sectional	C	362	23.89 ± 4.66	3 4 6 25	8
Tingting Lu	2023	Hypertension	China	Cross-sectional	A	432	3.83 ± 1.91	4 8 12 26 27 28 29	8
Zhiying shen	2020	Hypertension	China	Cross-sectional	C	790	23.83 ± 4.99	3 6 17 24	8
Zhuqing zhong	2016	Acute coronary syndrome	China	Cross-sectional	A	153	4.85 ± 1.52	1 3	7
Zhuqing zhong	2019	Hypertension	China	Cross-sectional	A	132	4.89 ± 1.28	1 3 30	7
Xiao chang	2023	Stroke	China	Cross-sectional	B	307	8.95 ± 3.49	3 6 19 31	8
Jiling qu	2021	Coronary heart disease	China	Cross-sectional	B	280	Inadequate medication literacy (%): 41.4	−	8
Feng zheng	2020	Coronary heart disease	China	Cross-sectional	B	470	7.52 ± 4.09	−	7
Guiyue ma	2020	Hypertension	China	Cross-sectional	C	540	24.61 ± 5.13	−	7
Xing ming	2026	Hypertension	China	Cross-sectional	D	171	37.0 (32.0, 42.0)	3 6 14	9
Sayekti	2018	Hypertension	Indonesia	Cross-sectional	G	109	2.35 ± 1.45	3	5

Measure tool: A: Medication Literacy Questionnaire (MLQ); B: Medication Literacy Scale in Spanish and English (MedLitRxSE); C: Chinese Medication Literacy Scale for Hypertensive Patients (C-MLSHP); D: Revised Chinese Medication Literacy Scale for Hypertensive Patients (C-MLSHP-R); E: Medication Literacy Scale for Older Adults with Chronic Diseases (MLS-OCD); F: Medication Literacy Questionnaire for Rural Elderly Patients with Cardiovascular Disease (MLQ-RECVD); G: Medication Label Instrument (MLI).‐: indicates not reported.

Influencing factor: 1. Age; 2. Gender; 3. Educational level; 4. Social support; 5. Exercise status; 6. Income level; 7. Hospitalized Department; 8. Self-efficacy; 9. Presence of Hypertension; 10. Presence of Diabetes; 11. Types of Medications Discharged; 12. Receipt of Medication Education; 13. Self-Care Ability; 14. Occupational Status; 15. Fasting Blood Glucose Level; 16. Current Residence; 17. Recent Medications; 18. Ability to Read Medication Instructions; 19. Family history of stroke; 20. Medical and social support; 21. Pharmacological beliefs; 22. Type of atrial fibrillation; 23. Hope level; 24. Disease duration; 25. Number of household members; 26. Blood pressure control status; 27. Use of community health education resources; 28. Marital status; 29. Number of annual medical visits; 30. Length of hospital stay; 31. Number of health problems.

### 3.3 Results of the meta-analysis

#### 3.3.1 Results of the meta-analysis of medication literacy scores

**FIGURE 2:**
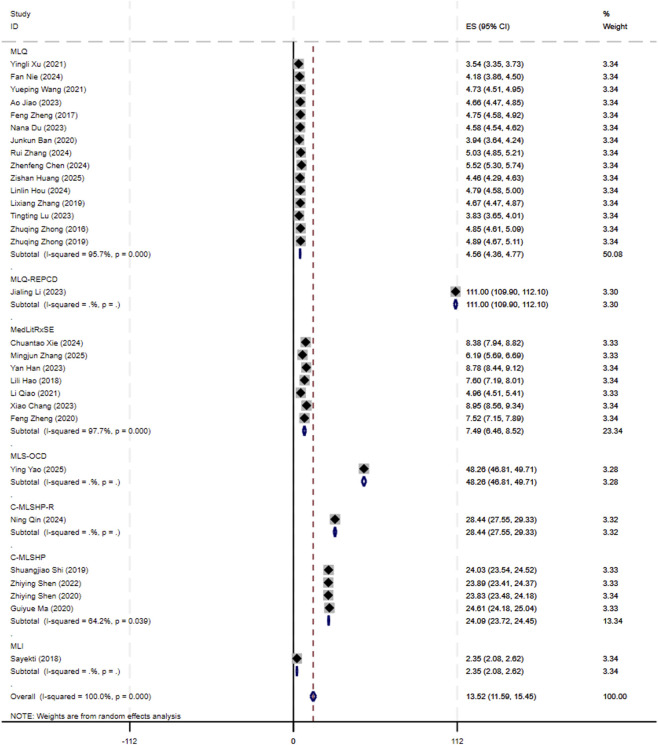
Forest plot of subgroup analysis based on medication literacy scores among patients with cardiovascular diseases.

Among the 34 included studies, medication literacy score data could not be extracted from four studies (Qu et al., 2021; Zhang et al., 2021; Ye, 2024; Ming et al., 2026); therefore, the meta-analysis was conducted on the remaining 30 studies. Fifteen studies (Zhong et al., 2016; Zhong et al., 2019; Zheng et al., 2017; Zhang et al., 2019; Zhang et al., 2024; Ban and Fa, 2020; Wang et al., 2021; Xu, 2021; Du and Xia, 2023; Jiao et al., 2023; Lu et al., 2023; Chen et al., 2024; Hou et al., 2024; Nie and Luo, 2024; Huang et al., 2025) used the Medication Literacy Questionnaire (MLQ). Significant heterogeneity was observed (I^2^ = 95.7%, P < 0.001). Sensitivity analysis showed that excluding any single study did not substantially affect the pooled estimate. Using a random-effects model, the pooled score was 4.56 (95% CI: 4.36, 4.77). Seven studies (Hao, 2018; Zheng et al., 2020; Qiao et al., 2021; Chang et al., 2022; Han et al., 2023; Xie et al., 2024; Zhang et al., 2025) used the Medication Literacy Scale in Spanish and English (MedLitRxSE). High heterogeneity was observed (I^2^ = 97.7%, P < 0.001). Sensitivity analysis indicated stable results. The pooled score was 7.49 (95% CI: 6.46, 8.52) using a random-effects model. Four studies (Shi et al., 2019; Ma et al., 2020; Shen et al., 2020; Shen et al., 2022) used the Chinese Medication Literacy Scale for Hypertensive Patients (C-MLSHP). Moderate heterogeneity was observed (I^2^ = 64.2%, P = 0.039). Sensitivity analysis showed stable results. The pooled score was 24.09 (95% CI: 23.72, 24.45) based on a random-effects model. The remaining four studies (Sayekti et al., 2018; Qin et al., 2024; Yao et al., 2025; Li et al., 2023) used different instruments, including the Revised Chinese Medication Literacy Scale for Hypertensive Patients (C-MLSHP-R), the Medication Literacy Scale for Older Adults with Chronic Diseases (MLS-OCD), the Medication Literacy Questionnaire for Rural Elderly Patients with Cardiovascular Disease (MLQ-RECVD), and the Medication Label Instrument (MLI). The corresponding scores were 28.44 (95% CI: 27.55, 29.33), 48.26 (95% CI: 46.81, 49.71), 111.00 (95% CI: 109.90, 112.10), and 2.35 (95% CI: 2.08, 2.62), respectively (Figure 2).

#### 3.3.2 Subgroup analysis of medication literacy scores

To explore potential sources of heterogeneity related to disease type and sample size, subgroup analyses were conducted stratified by measurement scale. For the C-MLSHP scale included only patients with hypertension and all studies had sample sizes >300, no subgroup analysis was performed for the C-MLSHP scale. For studies using the MLQ, subgroup analyses were conducted based on disease type, including hypertension, coronary heart disease, stroke, and atrial fibrillation. The pooled scores were 4.36 (95% CI: 3.32, 5.40), 4.41 (95% CI: 3.98, 4.85), 4.97 (95% CI: 4.58, 5.37), and 4.46 (95% CI: 4.21, 4.71), respectively. When stratified by sample size (≤300 vs. >300), the pooled scores were 4.58 (95% CI: 4.32, 4.85) and 4.43 (95% CI: 3.25, 5.61), respectively. No statistically significant differences were observed between subgroups (P > 0.05). For studies using the MedLitRxSE scale, subgroup analyses based on disease type (coronary heart disease, stroke, and others) yielded pooled scores of 6.23 (95% CI: 4.77, 7.68), 8.85 (95% CI: 8.60, 9.11), and 7.99 (95% CI: 7.22, 8.75), respectively. A statistically significant difference was observed across disease types (P < 0.05). When stratified by sample size (≤300 vs. >300), the pooled scores were 6.90 (95% CI: 5.52, 8.28) and 7.72 (95% CI: 6.29, 9.15), respectively, with no statistically significant difference between subgroups (P > 0.05).

#### 3.3.3 Results of the meta-analysis on factors influencing medication literacy

A total of 31 potential influencing factors were identified across the included studies, of which 11 were reported in at least two studies. These 11 factors were included in the meta-analysis. When pooling data for risk factors, if the classification criteria for a particular factor differed across studies, the studies with the most consistent classification criteria were selected for pooling. Heterogeneity assessment showed that four factors (hospitalized department, presence of hypertension, types of medications discharged, and receipt of medication education) exhibited low heterogeneity and were analyzed using a fixed-effects model. In contrast, seven factors (age, educational level, exercise status, income level, occupational status, social support, and self-efficacy) showed substantial heterogeneity and were analyzed using a random-effects model. The meta-analysis demonstrated that nine of the eleven factors were significantly associated with medication literacy (P < 0.05) (Tables 2, 3).

**TABLE 2:** Meta-analysis of factors influencing medication literacy among patients with cardiovascular diseases (continuous variables).

Influencing factors	Number of included studies (n)	Heterogeneity test		Effect model	Result of meta-analysis		
		I^2^ (%)	P		SMD (95% CI)	Z	P
Age (younger vs. older)	5	77.4%	0.001	Random	1.11 (0.78, 1.43)	6.60	<0.001
Educational attainment (≤ junior high school vs. ≥ high school)	13	88.4%	<0.001	Random	−1.01 (−1.24, −0.79)	8.76	<0.001
Exercise status (no vs. yes)	2	92.2%	<0.001	Random	−1.02 (−2.04, 0.00)	1.95	0.051
Income level	​	​	​		​	​	​
low-medium	9	75.5%	<0.001	Random	−0.41 (−0.60, −0.22)	4.22	<0.001
low-high	9	95.7%	<0.001	Random	−0.59 (−1.07, −0.11)	2.43	0.015
medium-high	9	93.9%	<0.001	Random	−0.26 (−0.71, 0.18)	1.15	0.249
Hospitalized department (general ward vs. CCU)	2	0%	0.995	Fixed	0.45 (0.17, 0.72)	3.18	0.001
Presence of hypertension (yes vs. no)	3	0%	0.491	Fixed	−0.64 (−0.86, −0.43)	5.85	<0.001
Types of medications discharged (fewer vs. more)	3	0%	0.375	Fixed	1.11 (0.88, 1.34)	9.60	<0.001
Receipt of medication education (yes vs. no)	3	0%	0.461	Fixed	0.83 (0.59, 1.07)	6.86	<0.001
Occupational status (employed vs. unemployed)	2	96.2%	<0.001	Random	0.72 (−0.46, 1.90)	1.20	0.229

**TABLE 3:** Meta-analysis of factors influencing medication literacy among patients with cardiovascular diseases (related variables).

Influencing factors	Number of included studies (n)	Heterogeneity test		Effect model	Result of meta-analysis		
		I^2^ (%)	P		Summary Fisher’s Z (95% CI)	P	Summary R (95% CI)
Social support	5	86.9%	<0.001	Random	0.56 (0.39, 0.73)	<0.001	0.51 (0.37, 0.62)
Self-efficacy	4	89.5%	<0.001	Random	0.56 (0.36, 0.76)	<0.001	0.51 (0.35, 0.64)

#### 3.3.4 Descriptive analysis

Several studies (Zhong et al., 2019; Zhang et al., 2019; Jiao et al., 2023; Lu et al., 2023; Hou et al., 2024; Nie and Luo, 2024; Huang et al., 2025; Hao, 2018; Qiao et al., 2021; Chang et al., 2022; Han et al., 2023; Shen et al., 2020; Shen et al., 2022; Yao et al., 2025; Li et al., 2023) also reported additional potential factors associated with medication literacy. Based on the HEM, Personal trait factors included female gender, comorbid diabetes, fasting blood glucose ≥7.1 mmol/L, family history of stroke, persistent atrial fibrillation, long disease duration, poor blood pressure control, hospital stays of ≤8 days, higher number of daily medications, and a higher number of health problems. Psychological and behavioral characteristics factors included inability to use medication leaflets, low belief in the necessity of medication, low levels of hope, and fewer annual clinic visits. Social networking factors included being single, low medical and social support, and living with ≥5 people. Work and living conditions factors included rural residence. In addition, difficulty in utilising community educational resources is the policy and environmental factors. These factors may be associated with lower levels of medication literacy; however, due to limited available data and inconsistent reporting across studies, only descriptive analysis was performed.

#### 3.3.5 Sensitivity analysis

Sensitivity analyses were conducted by comparing results obtained from fixed-effects and random-effects models to evaluate the robustness of the pooled effect sizes and their directions. The results indicated that, for most factors, the direction and statistical significance of the pooled effect sizes were consistent across models, indicating that the findings were robust. However, for certain variables, including exercise status and occupational status, substantial heterogeneity was observed, and the results differed between the two models, suggesting that these findings may be less stable. This may be attributable to the limited number of studies (n = 2 for each factor). Therefore, these results should be interpreted with caution, and further studies are needed to confirm their reliability (Table 4).

**TABLE 4:** Sensitivity analysis of factors influencing medication literacy among patients with cardiovascular diseases.

Influencing factors	Fixed effect model			Random effect model			Stability
	SMD/Summary r (95% CI)	I^2^ (%)	P	SMD/Summary r (95% CI)	I^2^ (%)	P	
Age^a^ (younger vs. older)	1.11 (0.97, 1.26)	77.4%	0.001	1.11 (0.78, 1.43)	77.4%	0.001	Stabilize
Educational attainment^a^ (≤ junior high school vs. ≥ high school)	−0.86 (−0.93, −0.79)	88.4%	<0.001	−1.01 (−1.24, −0.79)	88.4%	<0.001	Stabilize
Exercise status^a^ (no vs. yes)	−0.90 (−1.18, 0.62)	92.2%	<0.001	−1.02 (−2.04, 0.00)	92.2%	<0.001	Instability
Income level^a^							
low-medium	−0.33 (−0.42, −0.24)	75.5%	<0.001	−0.41 (−0.60, −0.22)	75.5%	<0.001	Stabilize
low-high	−0.68 (−0.78, −0.59)	95.7%	<0.001	−0.59 (−1.07, −0.11)	95.7%	<0.001	Stabilize
medium-high	−0.38 (−0.48.0.28)	93.9%	<0.001	−0.26 (−0.71, 0.18)	93.9%	<0.001	Stabilize
Hospitalized department^a^ (general ward vs. CCU)	0.45 (0.17, 0.72)	0%	0.995	0.45 (0.17, 0.72)	0%	0.995	Stabilize
Presence of hypertension^a^ (yes vs. no)	−0.64 (−0.86, −0.43)	0%	0.491	−0.64 (−0.86, −0.43)	0%	0.491	Stabilize
Types of medications discharged^a^ (fewer vs. more)	1.11 (0.88, 1.34)	0%	0.375	1.11 (0.88, 1.34)	0%	0.375	Stabilize
Receipt of medication education^a^ (yes vs. no)	0.83 (0.59, 1.07)	0%	0.461	0.83 (0.59, 1.07)	0%	0.461	Stabilize
Occupational status^a^ (employed vs. unemployed)	0.33 (0.16, 0.50)	96.2%	<0.001	0.72 (−0.46, 1.90)	96.2%	<0.001	Instability
Social support^b^	0.48 (0.44, 0.53)	86.9%	<0.001	0.51 (0.37, 0.62)	86.9%	<0.001	Stabilize
Self-efficacy^b^	0.49 (0.44, 0.53)	89.5%	<0.001	0.51 (0.35, 0.64)	89.5%	<0.001	Stabilize

^a^ SMD.

^b^ Summary r.

#### 3.3.6 Publication bias analysis

For factors with at least 10 included studies, publication bias was assessed using Egger’s test. The Egger’s test indicated potential publication bias for educational level (P = 0.037). The trim-and-fill method was subsequently applied to further evaluate its impact. The analysis indicated that no additional studies were imputed (imputed = 0), and the pooled effect sizes remained largely unchanged before and after adjustment (ES = −1.012, 95% CI: −1.228, −0.796). Therefore, although Egger’s test suggested potential bias, the trim-and-fill results indicate that the impact of publication bias on the overall results appears to be minimal, indicating that the findings are relatively robust.

## 4 Discussion

### 4.1 The current state of medication literacy among patients with CVDs

This study brings together findings on medication literacy among patients with CVDs using multiple measurement tools. Although the pooled results varied across scales, overall levels of medication literacy are generally inadequate, consistent with previous studies (Xie et al., 2022; Ni et al., 2025). This finding indicates substantial gaps in patients’ ability to understand medication-related information and make appropriate medication decisions, which may be related to the complexity of treatment regimens and the high prevalence of polypharmacy among this population (Sheikh-Taha and Asmar, 2021). Considerable variation was evident across different measurement scales, which may reflect differences in their theoretical frameworks, measurement domains, and scoring systems. Therefore, direct comparisons across scales should be interpreted with caution, and this variability points to the lack of a standardized assessment framework for medication literacy. Subgroup analyses further indicated significant differences in medication literacy scores across disease types when assessed using the MedLitRxSE scale, with higher scores observed among patients with stroke compared to those with coronary heart disease. One possible explanation may be that longer hospitalization periods and increased exposure to medication education are more common among patients with stroke. Overall, these results highlight the importance of improving medication literacy among patients with CVDs. Future research may benefit from the development of standardized assessment tools and more targeted approaches to improving patients’ medication management capabilities.

### 4.2 Analysis of factors influencing the current state of medication literacy among patients with CVDs

#### 4.2.1 Personal trait factors

The meta-analysis identified age as an important factor associated with medication literacy among patients with CVDs (P < 0.001), with younger patients tending to demonstrate higher levels of medication literacy, in line with earlier reports (Plaza-Zamora et al., 2020; Mortelmans et al., 2021; Deng et al., 2025). This relationship can be understood from multiple perspectives. From a physiological perspective, aging is associated with cognitive decline, potentially reducing the capacity to process and apply complex medication-related information (Lima et al., 2024). Behaviorally, older patients may be less likely to actively seek medication-related information and may rely more on caregivers or healthcare providers, which can restrict information uptake (Yuen et al., 2024). Moreover, multimorbidity and polypharmacy further complicate medication management in older populations (Mortelmans et al., 2024). Taken together, tailored medication education strategies are warranted for older patients, including simplified instructions, the use of visual aids, and greater involvement of caregivers.

The analysis also indicated hospital department as a significant factor (P < 0.05), with patients in general wards showing relatively higher levels of medication literacy than those in coronary care units (CCUs). This difference is likely associated with the more severe clinical conditions, higher treatment complexity, and greater psychological stress experienced by patients in CCUs, which are factors that have been linked to lower capacity to engage with medication-related information (Ye, 2024; Wang et al., 2021). At the same time, care in CCUs tends to prioritize acute care and monitoring, which may be associated with fewer opportunities for structured medication education (Tian et al., 2024). In response to this, it is recommended that healthcare systems consider implementing structured roles for specialized health education to provide individualized and continuous medication guidance for critically ill patients, without disrupting routine clinical workflows.

Furthermore, the presence of hypertension and a higher number of discharge medications were associated with lower levels of medication literacy (P < 0.001), consistent with findings from previous studies (Zhang et al., 2021; Bae et al., 2016; Zhong et al., 2018). This may be related to the increased complexity associated with polypharmacy, including complex dosing schedules and potential drug interactions, which have been linked to greater cognitive burden and lower levels of medication self-management (Mortelmans et al., 2024). Simplified and individualized medication management approaches, such as written medication lists and structured dosing schedules, may help address these challenges. Previous studies (Desteghe et al., 2018) also suggest that supportive tools, including smart pill organizers, medication reminder systems, and digital health applications, may facilitate medication management and adherence in patients with complex treatment regimens.

#### 4.2.2 Psychological and behavioral characteristics factors

The present study identified self-efficacy as a key determinant of medication literacy (P < 0.001), with lower self-efficacy levels being associated with poorer medication literacy. This finding is consistent with previous research (Sugiharto et al., 2024). Self-efficacy reflects an individual’s perceived capability to perform specific tasks (Shorey et al., 2021). Patients with low self-efficacy often report perceiving medication management as complex and difficult to control, and may be less likely to engage with health information or may rely more passively on healthcare providers, patterns that have been linked to lower medication literacy (Achury-Saldaña et al., 2025). Furthermore, according to Bandura’s self-efficacy theory (Bahn, 2001), self-efficacy can be enhanced through mastery experiences, vicarious experiences, and verbal persuasion. Accordingly, healthcare professionals can support patients by providing opportunities for hands-on practice, utilizing peer education to provide role-modeling experiences, and offering specific and positive feedback during routine care. These strategies may enhance patients’ self-efficacy in medication management, which has been associated with better medication literacy outcomes.

#### 4.2.3 Social networking factors

The pooled results indicated a significant positive association between social support and medication literacy among patients with cardiovascular disease (P < 0.001), consistent with previous findings (Chamberlain et al., 2024). Social support generally includes emotional and instrumental assistance from family members, caregivers, and social institutions (Mendonça et al., 2014), and is associated with higher medication literacy through multiple pathways. At a practical level, family members often help patients interpret medication instructions, adhere to dosing schedules, and manage medication-related problems. From a psychological perspective, adequate social support can enhance patients’ motivation for healthy behaviors and confidence in disease management (Gallant, 2003). Additionally, by alleviating anxiety, it may improve patients’ willingness and ability to engage with health education (DiMatteo, 2004). Consequently, healthcare professionals should involve family members or caregivers in medication education and implement family-participatory models. In addition, patient support groups or peer-support programs should be established to facilitate the sharing of experiences, consistent with evidence indicating that social support is positively associated with medication literacy.

#### 4.2.4 Work and living conditions factors

The analysis identified income level as a significant factor associated with medication literacy among patients with CVDs (P < 0.001), with higher income associated with better medication literacy, consistent with previous findings (Perpétuo et al., 2025). Higher income is generally associated with improved access to healthcare resources and health information, including medical consultations, health education, and digital health tools (Schillinger et al., 2002). It is also linked to more proactive health behaviors and stronger self-management capabilities (Hardman et al., 2020). Conversely, patients with lower income levels have been associated with poorer medication literacy, a pattern that may reflect financial constraints, infrequent healthcare utilization, and limited access to information (Ma et al., 2020). Consequently, healthcare professionals should assess patients’ financial status during discharge counseling and assist them in understanding health insurance reimbursement policies and covered medications. In addition, generic substitution strategies should be promoted to reduce long-term medication costs (Shrank et al., 2006). For patients experiencing financial hardship, medication subsidies may be provided through chronic disease management programs or drug assistance schemes to minimize treatment discontinuation and improve medication literacy.

The meta-analysis indicated that educational attainment is a significant determinant of medication literacy among patients with cardiovascular disease (P < 0.001), with higher educational attainment associated with better medication literacy, consistent with previous findings (Alkatheri and Albekairy, 2013). Patients with higher educational attainment generally demonstrate stronger abilities in information comprehension and processing, which may correlate with better understanding of medication instructions and health materials. In contrast, patients with lower educational attainment face substantial barriers in accessing, understanding, and evaluating medication-related information, often requiring assistance from healthcare professionals or caregivers (Ma et al., 2020; Sayekti et al., 2018). Therefore, healthcare professionals should assess patients’ educational levels and tailor communication using plain language, visual aids, or multimedia tools to improve medication literacy among individuals with lower educational attainment.

#### 4.2.5 Policy and environmental factors

The analysis showed that receipt of medication education is a significant factor influencing medication literacy among patients with CVDs (P < 0.001), with those receiving education demonstrating higher levels of medication literacy (Alkatheri and Albekairy, 2013). Receipt of structured medication education is associated with better patient understanding of key information, including drug names, mechanisms of action, administration methods, and potential adverse effects (Zhang et al., 2019; Jiao et al., 2023; Huang et al., 2025). It is also associated with fewer medication errors, likely through improvements in patient-provider communication (Kripalani et al., 2007). In contrast, patients without such education are more likely to experience incorrect dosing, missed doses, or inappropriate discontinuation. Therefore, healthcare professionals should adopt structured medication education approaches during hospitalization, providing staged and targeted explanations supported by visual aids or simplified instructions. Additionally, the “teach-back” method (Talevski et al., 2020) should be used to assess patient understanding and promptly correct misunderstandings, which may contribute to improved medication literacy.

Overall, the results of this study indicate that medication literacy among patients with CVDs is influenced by multiple factors across different ecological levels. Furthermore, these factors often interact across these levels. For example, low income (individual and socioeconomic levels) may be associated with limited access to medication education (environmental and policy levels), and this limited access may, in turn, be associated with reduced self-efficacy (psychological level) (Ma et al., 2020; Shen et al., 2020). These multi-level interrelationships suggest that, compared to single-level interventions, multi-level intervention strategies may be more effective in optimizing medication management and improving long-term health outcomes for this population.

Another important observation is that the majority of included studies were conducted in China, with only a limited number from other regions. This geographic concentration may be attributed to several factors. First, the concept of medication literacy is more frequently studied as an independent construct in China, whereas in many Western countries it is often incorporated into broader frameworks such as health literacy, medication adherence, or patient education (Gazmararian et al., 2010; Dinges et al., 2022). As a result, relevant studies from other regions may not have been identified using the specific search terms applied in this review. Second, commonly used medication literacy assessment tools, such as the MLQ and C-MLSHP, have primarily been developed and validated in Chinese populations, which may limit the inclusion of studies using different instruments in other settings (Zheng et al., 2015; Zhong et al., 2020). Third, differences in healthcare systems and research priorities may also play a role, with some countries emphasizing integrated care and patient education rather than isolating medication literacy as a distinct research focus (Crengle et al., 2018; Smylie et al., 2018). Therefore, the observed geographic distribution of studies does not necessarily indicate that medication literacy is not recognized globally, but rather reflects variations in conceptualization, measurement, and research emphasis across regions.

## 5 Research limitations

Several limitations should be considered when interpreting the findings of this study. Only studies published in Chinese and English were included, which may have introduced potential language bias and restricted the scope of the available evidence. In addition, standardized mean differences were used to pool effect sizes for influencing factors, and some analyses were based on a limited number of studies (n = 2–3), which may affect the stability of the estimates and increase the possibility of residual confounding. The included studies used different instruments to assess medication literacy. Although subgroup analyses were performed, differences in conceptual frameworks and scoring systems may still limit comparability across studies. Furthermore, due to the limited number of studies in each measurement tool subgroup, no meta-regression analysis was performed. Moreover, all included studies were cross-sectional, which restricts the ability to draw causal inferences. Most included studies were conducted in China, which may limit the generalizability of the findings to other populations or settings. Future studies should focus on benefiting from large-scale, high-quality studies using standardized measurement tools, as well as longitudinal or experimental designs to further explore causal relationships, underlying mechanisms, and the sources of heterogeneity.

## 6 Conclusion

This study demonstrates that medication literacy among patients with CVDs remains suboptimal and is shaped by multidimensional factors spanning individual, psychological, social, and environmental levels. Clinicians and pharmacists should deliver individualized medication education and proactive adherence support tailored to patients’ literacy levels. Healthcare systems and policymakers should strengthen standardized medication counseling protocols and expand community-based management services for high-risk populations. Professional training programs should systematically cultivate healthcare providers’ competencies in medication literacy assessment and patient communication. Future multilevel interventions grounded in the HEM hold promise for optimizing medication use, reducing adverse drug events, improving cardiovascular outcomes, and informing the development of evidence-based healthcare policies.

## Data Availability

The original contributions presented in the study are included in the article/Supplementary Material, further inquiries can be directed to the corresponding author.
