# Supplementary material for: The current status and influencing factors of medication literacy among patients with cardiovascular diseases: a systematic review and meta-analysis
Source: Front Pharmacol. 2026 Jun 3;17:1849302. doi: 10.3389/fphar.2026.1849302 (PMC13272478; doi:10.3389/fphar.2026.1849302)
Supplement: Supplementary file 1 [file Table1.docx]

Supplementary Material

# 1. Supplementary Material S1. PRISMA 2020 checklist

| **Section and Topic** | **Item #** | **Checklist item** | **Location where item is reported** |
| --- | --- | --- | --- |
| **TITLE** | | |  |
| Title | 1 | Identify the report as a systematic review. |  |
| **ABSTRACT** | | |  |
| Abstract | 2 | See the PRISMA 2020 for Abstracts checklist. |  |
| **INTRODUCTION** | | |  |
| Rationale | 3 | Describe the rationale for the review in the context of existing knowledge. |  |
| Objectives | 4 | Provide an explicit statement of the objective(s) or question(s) the review addresses. |  |
| **METHODS** | | |  |
| Eligibility criteria | 5 | Specify the inclusion and exclusion criteria for the review and how studies were grouped for the syntheses. |  |
| Information sources | 6 | Specify all databases, registers, websites, organisations, reference lists and other sources searched or consulted to identify studies. Specify the date when each source was last searched or consulted. |  |
| Search strategy | 7 | Present the full search strategies for all databases, registers and websites, including any filters and limits used. |  |
| Selection process | 8 | Specify the methods used to decide whether a study met the inclusion criteria of the review, including how many reviewers screened each record and each report retrieved, whether they worked independently, and if applicable, details of automation tools used in the process. |  |
| Data collection process | 9 | Specify the methods used to collect data from reports, including how many reviewers collected data from each report, whether they worked independently, any processes for obtaining or confirming data from study investigators, and if applicable, details of automation tools used in the process. |  |
| Data items | 10a | List and define all outcomes for which data were sought. Specify whether all results that were compatible with each outcome domain in each study were sought (e.g. for all measures, time points, analyses), and if not, the methods used to decide which results to collect. |  |
|  | 10b | List and define all other variables for which data were sought (e.g. participant and intervention characteristics, funding sources). Describe any assumptions made about any missing or unclear information. |  |
| Study risk of bias assessment | 11 | Specify the methods used to assess risk of bias in the included studies, including details of the tool(s) used, how many reviewers assessed each study and whether they worked independently, and if applicable, details of automation tools used in the process. |  |
| Effect measures | 12 | Specify for each outcome the effect measure(s) (e.g. risk ratio, mean difference) used in the synthesis or presentation of results. |  |
| Synthesis methods | 13a | Describe the processes used to decide which studies were eligible for each synthesis (e.g. tabulating the study intervention characteristics and comparing against the planned groups for each synthesis (item #5)). |  |
|  | 13b | Describe any methods required to prepare the data for presentation or synthesis, such as handling of missing summary statistics, or data conversions. |  |
|  | 13c | Describe any methods used to tabulate or visually display results of individual studies and syntheses. |  |
|  | 13d | Describe any methods used to synthesize results and provide a rationale for the choice(s). If meta-analysis was performed, describe the model(s), method(s) to identify the presence and extent of statistical heterogeneity, and software package(s) used. |  |
|  | 13e | Describe any methods used to explore possible causes of heterogeneity among study results (e.g. subgroup analysis, meta-regression). |  |
|  | 13f | Describe any sensitivity analyses conducted to assess robustness of the synthesized results. |  |
| Reporting bias assessment | 14 | Describe any methods used to assess risk of bias due to missing results in a synthesis (arising from reporting biases). |  |
| Certainty assessment | 15 | Describe any methods used to assess certainty (or confidence) in the body of evidence for an outcome. |  |
| **RESULTS** | | |  |
| Study selection | 16a | Describe the results of the search and selection process, from the number of records identified in the search to the number of studies included in the review, ideally using a flow diagram. |  |
|  | 16b | Cite studies that might appear to meet the inclusion criteria, but which were excluded, and explain why they were excluded. |  |
| Study characteristics | 17 | Cite each included study and present its characteristics. |  |
| Risk of bias in studies | 18 | Present assessments of risk of bias for each included study. |  |
| Results of individual studies | 19 | For all outcomes, present, for each study: (a) summary statistics for each group (where appropriate) and (b) an effect estimate and its precision (e.g. confidence/credible interval), ideally using structured tables or plots. |  |
| Results of syntheses | 20a | For each synthesis, briefly summarise the characteristics and risk of bias among contributing studies. |  |
|  | 20b | Present results of all statistical syntheses conducted. If meta-analysis was done, present for each the summary estimate and its precision (e.g. confidence/credible interval) and measures of statistical heterogeneity. If comparing groups, describe the direction of the effect. |  |
|  | 20c | Present results of all investigations of possible causes of heterogeneity among study results. |  |
|  | 20d | Present results of all sensitivity analyses conducted to assess the robustness of the synthesized results. |  |
| Reporting biases | 21 | Present assessments of risk of bias due to missing results (arising from reporting biases) for each synthesis assessed. |  |
| Certainty of evidence | 22 | Present assessments of certainty (or confidence) in the body of evidence for each outcome assessed. |  |
| **DISCUSSION** | | |  |
| Discussion | 23a | Provide a general interpretation of the results in the context of other evidence. |  |
|  | 23b | Discuss any limitations of the evidence included in the review. |  |
|  | 23c | Discuss any limitations of the review processes used. |  |
|  | 23d | Discuss implications of the results for practice, policy, and future research. |  |
| **OTHER INFORMATION** | | |  |
| Registration and protocol | 24a | Provide registration information for the review, including register name and registration number, or state that the review was not registered. |  |
|  | 24b | Indicate where the review protocol can be accessed, or state that a protocol was not prepared. |  |
|  | 24c | Describe and explain any amendments to information provided at registration or in the protocol. |  |
| Support | 25 | Describe sources of financial or non-financial support for the review, and the role of the funders or sponsors in the review. |  |
| Competing interests | 26 | Declare any competing interests of review authors. |  |
| Availability of data, code and other materials | 27 | Report which of the following are publicly available and where they can be found: template data collection forms; data extracted from included studies; data used for all analyses; analytic code; any other materials used in the review. |  |

*From:*  Page MJ, McKenzie JE, Bossuyt PM, Boutron I, Hoffmann TC, Mulrow CD, et al. The PRISMA 2020 statement: an updated guideline for reporting systematic reviews. BMJ 2021;372:n71. doi: 10.1136/bmj.n71. This work is licensed under CC BY 4.0. To view a copy of this license, visit <https://creativecommons.org/licenses/by/4.0/>

# 2. Supplementary Material S2. Database and Search Strategies

**PubMed**

(("Cardiovascular Diseases"[Mesh]) OR (Cardiovascular Disease[Title/Abstract] OR Disease, Cardiovascular[Title/Abstract] OR Cardiac Events[Title/Abstract] OR Cardiac Event[Title/Abstract] OR Event, Cardiac[Title/Abstract] OR Adverse Cardiac Event[Title/Abstract] OR Adverse Cardiac Events[Title/Abstract] OR Cardiac Event, Adverse[Title/Abstract] OR Cardiac Events, Adverse[Title/Abstract] OR Major Adverse Cardiac Events[Title/Abstract] OR Cerebrovascular Disease[Title/Abstract] OR Coronary Heart Disease[Title/Abstract] OR Heart failure[Title/Abstract] OR Heart disease[Title/Abstract] OR Myocardial Infarction[Title/Abstract] OR Atrial Fibrillation[Title/Abstract] OR Hypertension[Title/Abstract] OR Cardiac Insufficiency[Title/Abstract] OR Arrhythmia[Title/Abstract] OR Stroke[Title/Abstract])) AND (Medication Literacy[Title/Abstract] OR Drug Literacy[Title/Abstract] OR Pharmaceutical Literacy[Title/Abstract] OR Medication Knowledge[Title/Abstract] OR Medication Understanding[Title/Abstract] OR Prescription Understanding[Title/Abstract] OR Prescription Knowledge[Title/Abstract] OR Medication Attitude[Title/Abstract] OR Healthy Medication Behavior[Title/Abstract] OR Health Literacy[Title/Abstract]) ("Cardiovascular Diseases"[MeSH Terms] OR ("cardiovascular disease"[Title/Abstract] OR "disease cardiovascular"[Title/Abstract] OR "cardiac events"[Title/Abstract] OR "cardiac event"[Title/Abstract] OR "event cardiac"[Title/Abstract] OR "adverse cardiac event"[Title/Abstract] OR "adverse cardiac events"[Title/Abstract] OR (("cardiacs"[All Fields] OR "Heart"[MeSH Terms] OR "Heart"[All Fields] OR "Cardiac"[All Fields]) AND "event adverse"[Title/Abstract]) OR "cardiac events adverse"[Title/Abstract] OR "major adverse cardiac events"[Title/Abstract] OR "cerebrovascular disease"[Title/Abstract] OR "coronary heart disease"[Title/Abstract] OR "heart failure"[Title/Abstract] OR "heart disease"[Title/Abstract] OR "myocardial infarction"[Title/Abstract] OR "atrial fibrillation"[Title/Abstract] OR "Hypertension"[Title/Abstract] OR "cardiac insufficiency"[Title/Abstract] OR "Arrhythmia"[Title/Abstract] OR "Stroke"[Title/Abstract])) AND ("medication literacy"[Title/Abstract] OR "drug literacy"[Title/Abstract] OR "pharmaceutical literacy"[Title/Abstract] OR "medication knowledge"[Title/Abstract] OR "medication understanding"[Title/Abstract] OR "prescription understanding"[Title/Abstract] OR "prescription knowledge"[Title/Abstract] OR "medication attitude"[Title/Abstract] OR (("healthies"[All Fields] OR "healthy"[All Fields]) AND "medication behavior"[Title/Abstract]) OR "health literacy"[Title/Abstract])

**Cochrane Library**

#1 MeSH descriptor: [Cardiovascular Diseases] explode all trees

#2 (Cardiovascular Disease OR Disease, Cardiovascular OR Cardiac Events OR Cardiac Event OR Event, Cardiac OR Adverse Cardiac Event OR Adverse Cardiac Events OR Cardiac Event, Adverse OR Cardiac Events, Adverse OR Major Adverse Cardiac Events OR Cerebrovascular Disease OR Coronary Heart Disease OR Heart failure OR Heart disease OR Myocardial Infarction OR Atrial Fibrillation OR Hypertension OR Cardiac Insufficiency OR Arrhythmia OR Stroke):ti,ab,kw (Word variations have been searched)

#3 #1 or #2

#4 (Medication Literacy OR Drug Literacy OR Pharmaceutical Literacy OR Medication Attitude):ti,ab,kw (Word variations have been searched)

#5 #3 and #4

**Embase**

#1. ('cardiovascular disease'/exp OR 'cardiovascular disease') AND [embase]/lim

#2. ('angiocardiopathy':ti,ab,kw OR 'angiocardiovascular disease':ti,ab,kw OR 'cardiovascular complication':ti,ab,kw OR 'cardiovascular diseases':ti,ab,kw OR 'cardiovascular disorder':ti,ab,kw OR 'cardiovascular disturbance':ti,ab,kw OR 'cardiovascular lesion':ti,ab,kw OR 'cardiovascular syndrome':ti,ab,kw OR 'cardiovascular vegetative disorder':ti,ab,kw OR 'complication, cardiovascular':ti,ab,kw OR 'disease, cardiovascular':ti,ab,kw OR 'major adverse cardiovascular event':ti,ab,kw OR 'cardiovascular disease':ti,ab,kw) AND [embase]/lim

#3. #1 OR #2

#4. ('medication literacy':ti,ab,kw OR 'drug literacy':ti,ab,kw OR 'pharmaceutical literacy':ti,ab,kw OR 'medication knowledge':ti,ab,kw OR 'medication understanding':ti,ab,kw OR 'prescription understanding':ti,ab,kw OR 'prescription knowledge':ti,ab,kw OR 'medication attitude':ti,ab,kw OR 'healthy medication behavior':ti,ab,kw) AND [embase]/lim

#5. #3 AND #4

**Web of science**

1: Cardiovascular Diseases OR Cardiovascular Disease OR Disease, Cardiovascular OR Cardiac Events OR Cardiac Event OR Event, Cardiac OR Adverse Cardiac Event OR Adverse Cardiac Events OR Cardiac Event, Adverse OR Cardiac Events, Adverse OR Major Adverse Cardiac Events OR Cerebrovascular Disease OR Coronary Heart Disease OR Heart failure OR Heart disease OR Myocardial Infarction OR Atrial Fibrillation OR Hypertension OR Cardiac Insufficiency OR Arrhythmia OR Stroke (Title) OR Cardiovascular Diseases OR Cardiovascular Disease OR Disease, Cardiovascular OR Cardiac Events OR Cardiac Event OR Event, Cardiac OR Adverse Cardiac Event OR Adverse Cardiac Events OR Cardiac Event, Adverse OR Cardiac Events, Adverse OR Major Adverse Cardiac Events OR Cerebrovascular Disease OR Coronary Heart Disease OR Heart failure OR Heart disease OR Myocardial Infarction OR Atrial Fibrillation OR Hypertension OR Cardiac Insufficiency OR Arrhythmia OR Stroke (Abstract)

2: Medication Literacy OR Drug Literacy OR Pharmaceutical Literacy OR Medication Attitude (Title) OR Medication Literacy OR Drug Literacy OR Pharmaceutical Literacy OR Medication Attitude (Abstract)

3: #2 AND #1

**CNKI**

(Subject: Cardiovascular Diseases) OR (Article Abstract: Cardiovascular Diseases + Cerebrovascular Diseases + Coronary Heart Disease + Heart Failure + Heart Disease + Myocardial Infarction + Atrial Fibrillation + Hypertension + Cardiac Insufficiency + Arrhythmia + Stroke (Exact)) AND (Subject: Medication Literacy) OR (Article Abstract: Medication Literacy + Medication Use Literacy + Medication Health Literacy + Pharmaceutical Literacy + Drug Literacy (Exact))

**VIP**

((((((((( (( Title or Keywords = Cardiovascular Disease OR Title or Keywords = Cerebrovascular Disease) OR Title or Keywords = Coronary Heart Disease) OR Title or Keywords = Heart Failure) OR Title or Keywords = Cardiac Disease) OR Title or Keywords = Myocardial Infarction) OR Title or Keywords = Atrial Fibrillation) OR Title or Keywords = Hypertension) OR Title or Keywords = Cardiac Dysfunction) OR Title or Keywords = Arrhythmia) OR Title or Keywords = Stroke) AND ((((Title or Keywords = Medication Literacy OR Title or Keywords = Medication Competence) OR Title or Keywords = Medication Health Literacy) OR Title or Keywords = Pharmaceutical Literacy) OR Title or Keywords = Drug Literacy))

**Wan Fang**

Title or Keywords: (cardiovascular disease OR cerebrovascular disease OR coronary heart disease OR heart failure OR heart disease OR myocardial infarction OR atrial fibrillation OR hypertension OR cardiac dysfunction OR arrhythmia OR stroke) and Title or Keywords: (medication literacy OR medication use literacy OR medication health literacy OR pharmaceutical literacy OR drug literacy)

**CBM**

1) “Cardiovascular disease”[Common Field: Smart] OR “Cerebrovascular disease”[Common Field: Smart] OR “Coronary heart disease”[Common Field: Smart] OR “Heart failure”[Common Field: Smart] OR “Heart disease”[Common Field: Smart] OR “Myocardial infarction” [Common Fields: Smart] OR “Atrial Fibrillation”[Common Fields: Smart] OR ‘Hypertension’[Common Fields: Smart] OR “Cardiac Insufficiency”[Common Fields: Smart]

2) “Arrhythmia” [Common Field: Smart] OR “Stroke” [Common Field: Smart]

3) (Arrhythmia [Common Field: Smart] OR Stroke [Common Field: Smart]) OR (Cardiovascular Disease [Common Field: Smart] OR Cerebrovascular Disease [Common Field: Smart] OR Coronary Heart Disease [Common Field: Smart] OR Heart Failure [Common Fields: Smart] OR “Heart Disease”[Common Fields: Smart] OR “Myocardial Infarction”[Common Fields: Smart] OR “Atrial Fibrillation”[Common Fields: Smart] OR ‘Hypertension’[Common Fields: Smart] OR “Cardiac Insufficiency”[Common Fields: Smart])

4) “Medication literacy” [Common field: Smart] OR “Medication use literacy” [Common field: Smart] OR “Medication health literacy” [Common field: Smart] OR “Pharmacy literacy” [Common field: Smart] OR “Drug literacy” [Common field: Smart]

5) (“Medication literacy”[Common Field: Smart] OR “Medication use literacy”[Common Field: Smart] OR “Medication health literacy”[Common Field: Smart] OR “Pharmacy literacy”[Common Field: Smart] OR “Drug literacy” [Common Fields: Smart]) AND (“Arrhythmia”[Common Fields: Smart] OR “Stroke”[Common Fields: Smart]) OR (“Cardiovascular Disease”[Common Fields: Smart] OR “Cerebrovascular Disease”[Common Fields: Smart] OR “Coronary Heart Disease”[Common Fields: Smart] OR “Heart Failure” [Common Fields: Smart] OR “Heart Disease”[Common Fields: Smart] OR “Myocardial Infarction”[Common Fields: Smart] OR “Atrial Fibrillation”[Common Fields: Smart] OR ‘Hypertension’[Common Fields: Smart] OR “Heart Failure”[Common Fields: Smart]))

# 3. Supplementary Material S3. **Quality assessment data**

| **Study** | **①** | **②** | **③** | **④** | **⑤** | **⑥** | **⑦** | **⑧** | **⑨** | **⑩** | **⑪** | **Total score** |
| --- | --- | --- | --- | --- | --- | --- | --- | --- | --- | --- | --- | --- |
| Yingli Xu | Yes | Yes | Yes | Yes | No | No | No | Yes | No | Yes | No | 6 |
| Fan Nie | Yes | Yes | Yes | Yes | No | No | No | Yes | No | Yes | No | 6 |
| Yueping Wang | Yes | Yes | Yes | No | No | No | Yes | Yes | No | Yes | No | 6 |
| Jieli Ye | Yes | No | Yes | No | No | No | Yes | Yes | No | Yes | No | 5 |
| Ao Jiao | Yes | Yes | Yes | Yes | No | No | Yes | Yes | No | Yes | No | 7 |
| Jialing Li | Yes | Yes | No | No | Yes | Yes | Yes | Yes | Yes | Yes | No | 8 |
| Chuantao Xie | Yes | Yes | Yes | No | No | Yes | Yes | Yes | No | Yes | No | 7 |
| Feng Zheng | Yes | Yes | Yes | No | No | Yes | Yes | Yes | No | Yes | No | 7 |
| Nana Du | Yes | Yes | Yes | No | No | Yes | Yes | Yes | No | Yes | No | 7 |
| Junkun Ban | Yes | Yes | Yes | No | No | Yes | No | Yes | No | Yes | No | 6 |
| Rui Zhang | Yes | Yes | Yes | No | No | Yes | Yes | Yes | No | Yes | No | 7 |
| Zhenfeng Chen | Yes | Yes | Yes | No | No | Yes | No | Yes | No | No | No | 5 |
| Mingjun Zhang | Yes | Yes | Yes | No | No | Yes | No | Yes | No | Yes | No | 6 |
| Ying Yao | Yes | Yes | Yes | No | No | Yes | Yes | Yes | No | Yes | No | 7 |
| Zishan Huang | Yes | Yes | Yes | No | No | Yes | Yes | Yes | Yes | Yes | No | 8 |
| Yan Han | Yes | Yes | Yes | No | No | Yes | No | Yes | No | No | No | 5 |
| Kunxiu Zhang | Yes | Yes | Yes | No | No | Yes | Yes | No | Yes | Yes | No | 7 |
| Linlin Hou | Yes | Yes | Yes | No | No | Yes | Yes | Yes | Yes | Yes | No | 8 |
| Lixiang Zhang | Yes | Yes | Yes | No | No | Yes | Yes | Yes | Yes | Yes | No | 8 |
| Lili Hao | Yes | Yes | No | No | No | Yes | Yes | No | No | Yes | No | 5 |
| Ning Qin | Yes | Yes | Yes | No | No | Yes | Yes | Yes | Yes | Yes | No | 8 |
| Shuangjiao Shi | Yes | Yes | Yes | No | No | Yes | Yes | Yes | Yes | Yes | No | 8 |
| Li Qiao | Yes | Yes | Yes | No | No | Yes | No | Yes | Yes | No | No | 6 |
| Zhiying Shen | Yes | Yes | Yes | No | No | Yes | Yes | Yes | Yes | Yes | No | 8 |
| Tingting Lu | Yes | Yes | Yes | No | No | Yes | Yes | Yes | Yes | Yes | No | 8 |
| Zhiying Shen | Yes | Yes | Yes | No | No | Yes | Yes | Yes | Yes | Yes | No | 8 |
| Zhuqing Zhong | Yes | Yes | Yes | No | No | Yes | Yes | Yes | No | Yes | No | 7 |
| Zhuqing Zhong | Yes | Yes | Yes | No | No | Yes | Yes | Yes | No | Yes | No | 7 |
| Xiao Chang | Yes | Yes | Yes | No | No | Yes | Yes | Yes | Yes | Yes | No | 8 |
| Jiling Qu | Yes | Yes | Yes | No | No | Yes | Yes | Yes | Yes | Yes | No | 8 |
| Feng Zheng | Yes | Yes | Yes | No | No | Yes | Yes | Yes | No | Yes | No | 7 |
| Guiyue Ma | Yes | Yes | Yes | No | No | Yes | Yes | Yes | No | Yes | No | 7 |
| Xing Ming | Yes | Yes | Yes | No | No | Yes | Yes | Yes | Yes | Yes | Yes | 9 |
| Sayekti | Yes | Yes | Yes | No | No | Yes | No | Yes | No | No | No | 5 |

Note: ①Define the source of information (survey, record review); ②List inclusion and exclusion criteria for exposed and unexposed subjects (cases and controls) or refer to previous publications; ③Indicate time period used for identifying patients; ④Indicate whether or not subjects were consecutive if not population-based; ⑤Indicate if evaluators of subjective components of study were masked to other aspects of the status of the participants; ⑥Describe any assessments undertaken for quality assurance purposes (e.g.test/retest of primary outcome measurements); ⑦Explain any patient exclusions from analysis; ⑧Describe how confounding was assessed and/or controlled; ⑨If applicable,explain how missing data were handled in the analysis; ⑩Summarize patient response rates and completeness of data collection; ⑪Clarify what follow-up,if any,was expected and the percentage of patients for which incomplete data or follow-up was obtained.
